# Habitual tea consumption and postoperative delirium after total hip/knee arthroplasty in elderly patients: The PNDABLE study

**DOI:** 10.1002/brb3.2612

**Published:** 2022-05-12

**Authors:** Xu Lin, Xiao‐xuan Li, Rui Dong, Bin Wang, Yan‐lin Bi

**Affiliations:** ^1^ Department of Anesthesiology Qingdao Municipal Hospital Affiliated to Qingdao University Qingdao China; ^2^ Department of Anesthesiology Qingdao Women and Children's Hospital Qingdao China; ^3^ Department of Anesthesiology Drum Tower Hospital Affiliated to Nanjing University Medical School Nanjing China

**Keywords:** cholinergic nerve system, elderly, hip/knee arthroplasty, postoperative delirium, tea

## Abstract

**Purpose:**

To clarify the effects of habitual tea consumption on postoperative delirium (POD) in elderly patients undergoing total hip/knee arthroplasty.

**Patients and Methods:**

A prospective cohort study was carried out at Qingdao Municipal Hospital Affiliated to Qingdao University between June 2020 and June 2021. A total of 332 patients aged 65–85 years undergoing total hip/knee arthroplasty under combined spinal and epidural anesthesia were enrolled from the Perioperative Neurocognitive Disorder and Biomarker Lifestyle (PNDABLE) study in the final analysis, consisting of 168 patients with habitual tea consumption and 164 patients with infrequent tea consumption. The primary endpoint was the effects of habitual tea consumption on POD and the incidence of POD, which was assessed by the Confusion Assessment Method (CAM) twice daily during the first 7 postoperative days, and POD severity was measured by the Memorial Delirium Assessment Scale (MDAS). The secondary endpoints were the concentrations of caffeine and tea polyphenols in plasma and cerebrospinal fluid (CSF), which were detected by the enzyme‐linked immunosorbent assay.

**Results:**

POD occurred in 61 of 332 patients (18.37%), among whom 19 had habitual tea consumption (5.72%) and 42 had infrequent tea consumption (12.65%). Habitual tea consumption (odds ratio [OR] = 0.370, 95% confidence interval [CI]: 0.205–0.670, *P* = .001) was significantly associated with POD in the logistic analysis, and then after adjusting for age and American Society of Anesthesiologists (ASA) physical status (OR = 0.353, 95% CI: 0.190–0.655, *P* = .001). Furthermore, caffeine in *T*_0_ plasma (OR = 0.834, 95% CI: 0.752–0.924, *P* = .001), *T*_1_ plasma (OR = 0.818, 95% CI: 0.738–0.908, *P* < .001), and CSF (OR = 0.899, 95% CI: 0.820–0.984, *P* = .022) and tea polyphenols in *T*_0_ plasma (OR = 0.541, 95% CI: 0.416–0.704, *P* < .001), *T*_1_ plasma (OR = 0.477, 95% CI: 0.359–0.633, *P* < .001), and CSF (OR = 0.526, 95% CI: 0.397–0.696, *P* < .001) were associated with POD after adjusting for age and ASA physical status.

**Conclusion:**

Habitual tea consumption may be associated with a lower incidence of POD in elderly patients.

## Graphical abstract

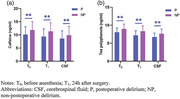

## 1 INTRODUCTION

Postoperative delirium (POD) is acute deterioration of brain function characterized by changes in mental state and attention, as well as disturbance of consciousness (Duque et al., 2018), and the main clinical manifestations of POD are fluctuating levels of consciousness and disorientation after surgery (Inouye et al., 2014). The cognitive function for self‐care ability of patients decreased (Hshieh et al., 2017). Clinical subtyping of delirium according to motor activity is widely used. Hypoactive type is featured by a decreased amount of activity, unawareness, and a decreased amount of speech, while patients with hyperactive type show agitation, irritability, and hallucination. POD is accompanied by the deterioration of prognosis: increased risk of short‐term complications, decreased long‐term cognitive function, the prolongation of hospital stay, and increased mortality (Oh & Park, 2019; Rudolph et al., 2008; Schenning & Deiner, 2015). However, at the current time, the pathophysiology of POD remains uncertain, and POD is associated with diverse mechanisms, including cholinergic deficiency, inflammatory response, abnormal energy metabolism, and the stress response. Studies have shown that cholinergic deficiency accounts for the majority of POD (Hshieh et al., 2008; Young et al., 1997).

Tea is among the most consumed beverages in the world. It is rich in both caffeine and tea polyphenols. Tea drinking originated in China around 4000–5000 years ago, and spread to the rest of the world. Caffeine is a central nervous system stimulant, which is naturally found in coffee, cocoa beans, and tea (Heckman et al., 2010). It is a unique compound that can be dissolved in water and nonpolar organic solvents, and distributed rapidly and efficiently throughout the whole body (including the brain) (Kolahdouzan & Hamadeh, 2017). It is generally believed that tea polyphenols, the main substance in tea, are good for health. The tea polyphenols can protect neurons via inhibition of dopamine (DA) oxidation, conjugation with dopamine quinones (DAQ), scavenge of reactive oxygen species (ROS), inhibition of monoamine oxidase B (MAOB), and modulations of nuclear factor erythroid 2‐related factor 2 (Nrf2)‐Keap1 and proliferator‐activated receptor gamma coactivator 1‐alpha (PGC‐1α) antioxidative signaling pathways, which have strong neuroprotective functions (Zhou et al., 2019). Some studies have found that caffeine and tea polyphenols exert neuroprotective effects, which can ameliorate cognitive impairment (Abreu et al., 2011; Çakır et al., 2017; Schimidt et al., 2017). Neuroprotection may play a role in the treatment of neuroinflammatory diseases (Venigalla et al., 2016). Namely, caffeine and tea polyphenols exert influence on the inflammatory response of POD via neuroprotective effects. Then, can caffeine and tea polyphenols influence neurocognitive functions through the cholinergic nervous system?

The vagus nerve stimulates the release of acetylcholine (ACh) via activating the cholinergic pathway, which plays a crucial role in cognitive function. Surgical trauma stimulates the innate immune system to release proinflammatory cytokines and, in particular, interleukin‐6 (IL‐6) and tumor necrosis factor‐α (TNF‐α). Increased proinflammatory cytokines can be transmitted to the brain and result in neuroinflammation, leading to blood–brain barrier (BBB) disruption. Central ACh can inhibit the inflammatory response by combining with α7‐nicotinic ACh receptors, which reduces BBB destruction and promotes brain protection (Naicker et al., 2016). The study has shown that α7‐nicotinic ACh receptors antagonism is involved in the formation of POD in aged rats with tibia fractures (Lin et al., 2019). With the increase of age, especially in the elderly, the cholinergic nervous system degenerates. The level of central ACh involved in learning and memory is reduced, which affects cognitive function, and the degradation of cholinergic nervous system is involved in the development of POD (Li et al., 2019; Xu et al., 2019).

In other words, it is important to increase the content of Ach, because enhancement of cholinergic neurotransmission may potentially reduce adverse outcomes of POD. There is a dynamic balance between ACh content in plasma and cerebrospinal fluid (CSF) under the interaction of choline acetyltransferase and cholinesterase predominantly including acetylcholinesterase (AChE) and butyrylcholinesterase (BuChE) that can efficiently and rapidly degrade ACh (Vijayaraghavan et al., 2013). Cholinesterase inhibitors increase central ACh levels by prolonging the residence time of ACh in the synaptic cleft, thereby enhancing cholinergic neurotransmission (Okello et al., 2012). An experimental animal study proved that caffeine could inhibit cholinesterase in rat brain (Akomolafe et al., 2017). Another study demonstrated that tea polyphenols could inhibit AChE and BuChE, as well as enhance the cholinergic neurotransmission (Ali et al., 2016). Furthermore, tea polyphenols can play a part in inhibiting cholinesterase activity in vitro and in vivo (Adsersen et al., 2006; Jessen et al., 2014; Kulišić‐Bilušić et al., 2008).

Therefore, we speculate that caffeine and tea polyphenols, as cholinesterase inhibitors, can reduce cholinesterase activity and ameliorate cognitive impairment. Our study explored neurodegenerative disease from the perspective of diet and lifestyle. Tea consumption is an effective, safe, and appropriate non‐pharmacological therapy for older people as well as a promising nonpharmacological intervention for patients with neurodegenerative diseases such as POD. Furthermore, we simultaneously analyzed plasma and CSF samples to evaluate their relationship.

Up to today, empirical evidence is still sparse and the value of tea drinking in the prevention of cognitive decline and POD is far from being firmly established. We aimed to prospectively examine the effects of habitual tea consumption on POD in Chinese elderly.

## 2 MATERIALS AND METHODS

### 2.1 The PNDABLE study

Participants were recruited from the Perioperative Neurocognitive Disorder and Biomarker Lifestyle (PNDABLE) study, which is a large cohort study conducted in 2018 to analyze the risk factors and biomarkers of perioperative neurocognitive impairment in the Han population in northern China for early diagnosis and prevention of the disease. The study has been registered with the China Clinical Trial Registry (clinical registration number: ChiCTR2000033439) and ethical approval for this study (Ethical Committee No. 2020 PRO FORMA Y number 005) was provided by the Ethical Committee Qingdao Municipal Hospital affiliated to Qingdao University, Qingdao, China (Chairman Prof Yang) on May 21, 2020. All patients were informed of the purpose of participation in the study and of the procedure (blood and CSF collection) and had signed an informed consent form prior to inclusion. Preoperative cognitive function was evaluated by Subjective Cognitive Decline Scale (SCDS), Mini‐Mental State Examination (MMSE), and Montreal Cognitive Assessment (MoCA); the patients were diagnosed as normal cognitive (NC), subjective cognitive decline (SCD), which was diagnosed with Jessen's criteria (Nasreddine et al., 2005), mild cognitive impairment (MCI), which was diagnosed with Nasreddine's criteria (McKhann et al., 2011), and Alzheimer's Disease (AD), which was diagnosed with Guy's criteria (Okello et al., 2004), according to the different criteria.

### 2.2 Participants

The study was conducted at Qingdao Municipal Hospital affiliated to Qingdao University between June 2020 and June 2021. We selected eligible patients who were aged 65–85 years and were scheduled to have total hip/knee arthroplasty under combined spinal and epidural anesthesia. The inclusion criteria of this study include (1) age 65–85 years; (2) Han Nationality Patients in north China; (3) American Society of Anesthesiologists (ASA) score Ⅰ or Ⅱ; (4) preoperative cognitive status was good with no language communication disorder; and (5) educational level was enough to complete preoperative cognitive function test. Patients were excluded if they had (1) taken caffeine‐containing drugs for more than 6 months or 1 week before surgery; (2) drunk coffee (including cocoa), soft drinks, milk tea, and eaten chocolate for more than 6 months; (3) preexisting neuropsychiatric disorders and the MMSE scores of 23 or less; (4) inability to read or severe visual or auditory deficits; (5) unwillingness to comply with the protocol and procedures; (6) preexisting anemia, electrolyte disorder, alcoholism, fever, hypoalbuminemia, and malnutrition; and (7) ASA score (a global score that assesses the physical status of patients before surgery, ranging from Ⅰ [normal health] to V [moribund]) greater than Ⅱ.

**FIGURE 1:**
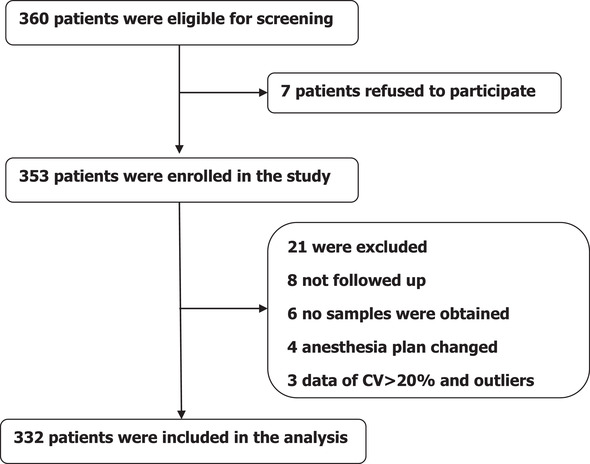
Patients screening and enrollment in the study. Depicted is the study flow diagram, including data on patients who were screened, enrolled, and excluded. CV, coefficient of variation; *T*_0_, before anesthesia; *T*_1_, 24 h after surgery.

On the basis of inclusion and exclusion criteria, a total of 360 participants were screened for our study, of whom 28 were excluded for various reasons and 332 participants were analyzed in our study (Figure 1).

### 2.3 Study protocol

This is a prospective cohort study. The MMSE was administered the day before surgery. According to frequency of tea consumption, participants were categorized into habitual tea consumption group (T group) and infrequent tea consumption group (NT group). At the same time, data collected at baseline included sociodemographic variables such as age, gender, height, body weight, knee/hip arthroplasty, ASA physical status, anesthetic time (the time that the anesthesiologists started the spinal anesthesia in the patients to the time that the patients were sent to the post‐anesthesia care unit), and operative time (the time of initial incision to the time of the closure of the skin). All the participants received combined spinal and epidural anesthesia meanwhile with dexmedetomidine sedation. There was no anyone received general anesthesia or never block for postoperative analgesia. The same patient‐controlled intravenous analgesia (PCIA) was used postoperatively for 48 h by all patients. The samples of peripheral blood (5 ml elbow vein blood) were collected at *T*_0_ (before anesthesia) and *T*_1_ (24 h after surgery). A sample of CSF (2 ml) was collected under spinal anesthesia. Participants were divided into POD group (P group) and non‐POD group (NP group).

### 2.4 Habitual tea consumption

Data on tea consumption were obtained using a validated self‐reported questionnaire including items like “whether you drink tea or not” and the frequency of tea consumption. Data on tea consumption were collected based on local tea drinking customs and terms such as “green tea,” “black tea,” and “oolong tea.” We divided the frequencies of tea consumption into the following categories: (1) never or <1 time/week; (2) 1–3 times/week; (3) >3 times/week; and (4) almost every day (Ma et al., 2020; Yasutake et al., 2016). A participant was classified as an infrequent tea consumer (NT group) if he/she fell into the Category (1) or (2); a participant was classified as a frequent tea consumer (T group) if he/she fell into the Category (3) or (4).

### 2.5 Anesthesia and surgery

All the participants underwent total hip/knee arthroplasty under combined spinal and epidural anesthesia by the same surgery team. Five‐lead electrocardiography, pulse oxygen saturation, and noninvasive blood pressure were monitored continuously during anesthesia and were recorded at fixed intervals of 5 min. The patients inhaled oxygen through the mask at 5 L/min, and the fluctuation of noninvasive blood pressure was kept within ±20% of the baseline value. However, glucocorticoid drugs, nonsteroidal analgesics, and midazolam were avoided during surgeries.

Postoperatively, the visual analogue scale score of 0–10 was used to assess pain in the patients at the same time. PCIA was used postoperatively for 48 h by all patients. The PCIA opioid consisted of 2.5 μg/kg sufentanil and 5 mg tropisetron (total volume of 100 ml, including 0.9% normal saline, bolus 2 ml, basal rate 2 ml/h, and lockout time 15 min). If patients need it, they were given nonopioid drugs for analgesia, which was recorded.

### 2.6 Sample collection and laboratory testing

Elbow vein blood samples (5 ml) were collected in an EDTA anticoagulant tube from the enrolled patients at *T*_0_ and *T*_1_. The CSF (2 ml) was collected in an RNase‐free Eppendorf tube during spinal anesthesia prior to the administration of the local anesthetics. The samples were immediately centrifuged at 2000 rpm for 10 min to remove cells, and the supernatant of blood sample (about 2 ml) and CSF sample (about 2 ml) was stored at −80°C until further analysis (Teunissen et al., 2009; Vanderstichele et al., 2012).

The levels of caffeine and tea polyphenols in plasma and CSF were measured by an enzyme‐labeling instrument (PerkinElmer, EnSpire, Waltham, MA, USA). The concentrations of AChE, BuChE, TNF‐α, and IL‐6 in plasma and CSF at *T*_0_ were measured by an enzyme‐labeling instrument (PerkinElmer, EnSpire). The activities of AChE and BuChE in plasma and CSF at *T*_0_ were measured by spectrometer (NanoDrop Technologies, Wilmington, DE, USA). All samples were assayed by the same laboratory assistant who was blinded to the group assignment.

### 2.7 Neuropsychological testing

The MMSE was administered by a neurologist 1 day before the operation, which is widely used as a screening tool for dementia and as a measure of global cognitive function. POD was defined by the Confusion Assessment Method (CAM) (Leung et al., 2008), and POD severity was measured using the Memorial Delirium Assessment Scale (MDAS) (Fabbri et al., 2001) at 10:00 a.m. and 2:00 p.m. twice a day on 1–7 days (or before discharge) by an anesthesiologist postoperatively. The above assessments performed by a neurologist and an anesthesiologist were not involved in intraoperative management of the patients. The CAM is the international standard for diagnosis of POD, including the following delirium characteristics: (1) acute onset with a fluctuating course; (2) inattention; (3) disordered thinking; and (4) changes in the level of consciousness (any state of consciousness other than fully conscious). The diagnosis of delirium requires the presence of (1) and (2), accompanied by (3) or (4) or both. The diagnosis of POD was performed by three doctors who had undergone training to ensure consistency.

### 2.8 Sample size estimation

Demographic and perioperative characteristics (including age and ASA physical status) were chosen as the independent variables and subjected to univariate logistic regression analysis. And then we controlled for age and ASA physical status due to their known effects on cognitive status (Krell‐Roesch et al., 2019).

The preliminary test in this study found that six covariates (plasma caffeine [*T*_0_], plasma tea polyphenols [*T*_0_], plasma caffeine [*T*_1_], plasma tea polyphenols [*T*_1_], CSF caffeine, and CSF tea polyphenols) were expected to enter the logistic regression, the POD incidence in preliminary test was 18.5%, and the loss of follow‐up rate was assumed to be 10%, so the required sample size was calculated to be 360 cases (6 × 10 ÷ 0.185 ÷ 0.9 = 360).

### 2.9 Statistical analysis

IBM SPSS software version 21.0 (SPSS, Inc., Chicago, IL, USA) and GraphPad prism software version 8.0 (GraphPad Software, San Diego, CA, USA) were used to analyze the data. All *P‐*values were two‐sided, and *P* < .05 was considered statistically significant. Continuous variables are expressed as arithmetic mean ± standard deviation (SD), whereas the median and interquartile range (IQR, 25–75 percentile) or a number (%) was used to express the data. The distribution of data was evaluated using the Kolmogorov–Smirnov test. The independent samples *t*‐test or Mann–Whitney *U* test was used to compare continuous variables, and chi‐square test was used to analyze categorical data.

The logistic regression was used to assess the associations between biomarkers of tea (independent variable *X*) and tea consumption (dependent variable *Y*). We adjusted possible confounding variables (age, gender, height, body weight, years of education, and body mass index [BMI]) associated with tea consumption. To assess the relationship between frequent tea consumption (independent variable *X*) and POD (dependent variable *Y*), demographic and perioperative characteristics (age, gender, height, body weight, BMI, years of education, knee arthroplasty, MMSE score, ASA physical status, anesthetic time, operative time, VAS score, habitual tea consumption, and biomarkers of tea) with POD chosen as the independent variables were subjected to univariate logistic regression analysis, and then the variables with *P* < .05 (including age and ASA physical status as adjusted factors) were established to be included in the multivariate logistic regression analysis. The multicollinearity was assessed using tolerance and variance inflation factor (VIF), and no multicollinearity existed in each model of the current study.

Spearman's rank correlation was used to determine the associations between the biomarkers (caffeine, tea polyphenols, AChE, BuChE, IL‐6, and TNF‐α) in preoperative plasma and CSF. Simultaneously, we explored the correlations of preoperative plasma biomarkers of tea (caffeine, tea polyphenols) with AChE, BuChE, IL‐6, and TNF‐α in preoperative plasma by Spearman's rank correlation.

## 3 RESULTS

### 3.1 Participants’ demographic characteristics

We screened 360 participants, of whom 28 participants were excluded from the study. And the reasons for dropouts are shown in Figure 1. Therefore, 332 patients remained for analysis. Except for age (*P* < .001) and ASA physical status (*P* < .001), there were no significant differences in terms of height, body weight, BMI, males, years of education, knee/hip arthroplasty, MMSE score, operative time, anesthetic time, and VAS scores between P group and NP group (*P* > .05). Additionally, there were significant differences in MDAS scores in the P group (13 [10–15]) compared with NP group (3 [1–5], *P* < .01) (Table 1).

**TABLE 1:** Demographic and perioperative characteristics between postoperative delirium group (P group) and non‐postoperative delirium group (NP group)

Variables	NP group (*N* = 271)	P group (*N* = 61)	*P*‐value
Age (years)	74.18 ± 6.05	77.38 ± 6.24	<.01
Height (cm)	165.82 ± 7.57	166.56 ± 7.49	.493
Body weight (kg)	68.34 ± 9.78	67.60 ± 7.37	.506
BMI (kg/m^2^)	24.90 ± 3.57	24.44 ± 2.64	.249
Males (%)	113 (41.70)	33 (54.10)	.078
Years of education, *n* (%)			
0	8 (2.95)	3 (4.92)	.203
1–9	116 (42.80)	29 (47.54)	.515
10–13	56 (20.66)	16 (26.23)	.382
14–17	78 (28.78)	8 (13.11)	.271
>17	13 (4.80)	5 (8.20)	.376
Knee arthroplasty (%)	182 (67.16)	33 (54.10)	.054
MMSE score	25.91 ± 1.02	25.62 ± 1.13	.054
ASA physical status (%)			
I	59 (21.77)	16 (26.23)	<.01
II	212 (78.23)	45 (73.77)	<.01
Anesthetic time^a^ (min)	150.74 ± 19.69	150.08 ± 17.26	.810
Operative time^b^ (min)	120.27 ± 21.08	114.84 ± 21.13	.070
Plasma caffeine (ng/ml) (*T*_0_)	11.84 ± 3.22	10.14 ± 2.93	<.01
Plasma tea polyphenols (ng/ml) (*T*_0_)	8.99 ± 1.34	8.04 ± 1.20	<.01
Plasma caffeine (ng/ml) (*T*_1_)	11.33 ± 3.29	9.38 ± 2.89	<.01
Plasma tea polyphenols (ng/ml) (*T*_1_)	8.33 ± 1.47	7.17 ± 1.29	<.01
CSF caffeine (ng/ml)	9.88 ± 3.38	8.60 ± 2.96	.007
CSF tea polyphenols (ng/ml)	7.64 ± 1.29	6.73 ± 1.07	<.01
Postoperative the highest MDAS score, median, and 25–75 percentile	3 (1–5)	13 (10–15)	<.01
Postoperative the highest VAS score, median, and 25–75 percentile	2 (1–3)	3 (2–5)	.654

Abbreviations: ASA, American Society of Anesthesiologists; BMI, body mass index; CSF, cerebrospinal fluid; MDAS, Memorial Delirium Assessment Scale; MMSE, China‐Modified Mini‐mental State Examination; NP, non‐POD; P, POD; SD, standard deviation; *T*_0_, before anesthesia; *T*_1_, 24 h after surgery; VAS, visual analog scale.

*Note*: Continuous variables are presented as mean and standard deviation, and categorical variables as number (percentage). All *P*‐values were calculated based on *t*‐test, Mann–Whitney *U* test, and chi‐square test.

^a^ The length of anesthesia was defined as from the time that the anesthesiologists started the spinal anesthesia in the patients to the time that the patients were sent to the postanesthesia care unit.

^b^ The length of surgery was defined from the time of initial incision to the time of the closure of the skin.

### 3.2 Primary outcomes

POD occurred in 61 of 332 patients (18.37%), among whom 19 had habitual tea consumption (5.72%) and 42 had infrequent tea consumption (12.65%).

### 3.3 Secondary outcomes

#### 3.3.1 Associations between tea consumption and biomarkers of tea in plasma and CSF

**FIGURE 2:**
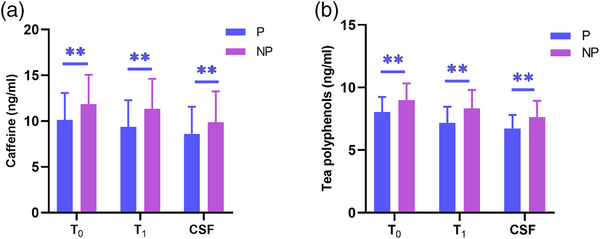
The concentrations of tea biomarkers in plasma and cerebrospinal fluid between P group and NP group. Comparison of the concentrations of caffeine (a) and tea polyphenols (b) at *T*_0_ and *T*_1_ in plasma and in CSF between P group and NP group. Standard deviations were calculated based on Mann–Whitney *U* test (**P < .01). CSF, cerebrospinal fluid; NP, non‐postoperative delirium; P, postoperative delirium; *T*_0_, before anesthesia; *T*_1_, 24 h after surgery.

After adjusting confounding variables (age, gender, height, body weight, and BMI) associated with tea consumption, caffeine in *T*_0_ plasma (odds ratio [OR] = 2.024, 95% confidence interval [CI]: 1.751–2.340, *P* < .001), *T*_1_ plasma (OR = 2.156, 95% CI: 1.840–2.527, *P* < .001), and CSF (OR = 1.607, 95% CI: 1.448–1.785, *P* < .001) was significantly associated with habitual tea consumption, and tea polyphenols in *T*_0_ plasma (OR = 1.496, 95% CI: 1.231–1.753, *P* < .001), *T*_1_ plasma (OR = 2.523, 95% CI: 1.988–3.201, *P* < .001), and CSF (OR = 1.577, 95% CI: 1.303–1.909, *P* < .001) were significantly associated with habitual tea consumption (Table 2). Of note, the concentrations of caffeine and tea polyphenols at *T*_0_ and *T*_1_ in P group were significantly lower than those in NP group (*P* < .05) (Figure 2).

**TABLE 2:** The associations between biomarkers in plasma and cerebrospinal fluid (CSF) (independent variable *X*) and tea consumption (dependent variable *Y*)

Biomarkers (ng/ml)	OR	95% CI	*P*‐value
Plasma caffeine (*T*_0_)	2.024	1.751–2.340	<.001
Plasma tea polyphenols (*T*_0_)	1.469	1.231–1.753	<.001
Plasma caffeine (*T*_1_)	2.156	1.840–2.527	<.001
Plasma tea polyphenols (*T*_1_)	2.523	1.988–3.201	<.001
CSF caffeine	1.607	1.448–1.785	<.001
CSF tea polyphenols	1.577	1.303–1.909	<.001

Abbreviations: CI, confidence interval; CSF, cerebrospinal fluid; OR, odds ratio; *T*_0_, before anesthesia; *T*_1_, 24 h after surgery.

#### 3.3.2 Associations of POD with tea consumption and biomarkers in plasma and CSF

In univariate logistic regression analyses (including demographic and perioperative characteristics), habitual tea consumption (OR = 0.370, 95% CI: 0.205–0.670, *P* = .001) was significantly associated with POD, and then after adjusting for age and ASA physical status (OR = 0.353, 95% CI: 0.190–0.655, *P* = .001) in multivariate logistic regression analysis. Caffeine in *T*_0_ plasma (OR = 0.834, 95% CI: 0.752–0.924, *P* = .001), *T*_1_ plasma (OR = 0.818, 95% CI: 0.738–0.908, *P* < .001), and CSF (OR = 0.899, 95% CI: 0.820–0.984, *P* = .022) and tea polyphenols in *T*_0_ plasma (OR = 0.541, 95% CI: 0.416–0.704, *P* < .001), *T*_1_ plasma (OR = 0.477, 95% CI: 0.359–0.633, *P* < .001), and CSF (OR = 0.526, 95% CI: 0.397–0.696, *P* < .001) were also significantly associated with POD after adjustment for age and ASA physical status (Table 3).

**TABLE 3:** The associations between biomarkers in plasma and cerebrospinal fluid (CSF) (independent variable *X*) and POD (dependent variable *Y*)

Biomarkers (ng/ml)	Unadjusted			Adjusted		
	OR	95% CI	*P*‐value	OR	95% CI	*P*‐value
Habitual tea consumption	0.370	0.205–0.670	.001	0.353	0.190–0.655	.001
Plasma caffeine (*T*_0_)	0.833	0.754–0.919	<.001	0.834	0.752–0.924	.001
Plasma tea polyphenols (*T*_0_)	0.542	0.421–0.697	<.001	0.541	0.416–0.704	<.001
Plasma caffeine (*T*_1_)	0.813	0.736–0.899	<.001	0.818	0.738–0.908	<.001
Plasma tea polyphenols (*T*_1_)	0.483	0.367–0.636	<.001	0.477	0.359–0.633	<.001
CSF caffeine	0.885	0.810–0.968	.007	0.899	0.820–0.984	.022
CSF tea polyphenols	0.508	0.385–0.669	<.001	0.526	0.397–0.696	<.001

*Note*: The demographic and perioperative characteristics were included in univariate logistic regression model. The multivariable logistic regression model was adjusted for age and ASA physical status.

Abbreviations: CI, confidence interval; CSF, cerebrospinal fluid; OR, odds ratio; POD, postoperative delirium; *T*_0_, before anesthesia; *T*_1_, 24 h after surgery.

#### 3.3.3 Correlations between CSF biomarkers and preoperative plasma biomarkers

Spearman's rank correlation indicated that the concentrations of caffeine (*r* = .812, *P* < .01), tea polyphenols (*r* = .836, *P* < .01), AChE (*r* = .862, *P* < .01), BuChE (*r* = .802, *P* < .01), and TNF‐α (*r* = .802, *P* < .01) as well as BuChE activity (*r* = .908, *P* < .01) in plasma were significantly associated with those in CSF; IL‐6 concentration (*r* = .727, *P* < .01) and AChE activity (*r* = .781, *P* < .01) in plasma were associated with those in CSF.

#### 3.3.4 Associations between preoperative plasma biomarkers of tea and other biomarkers

**FIGURE 3:**
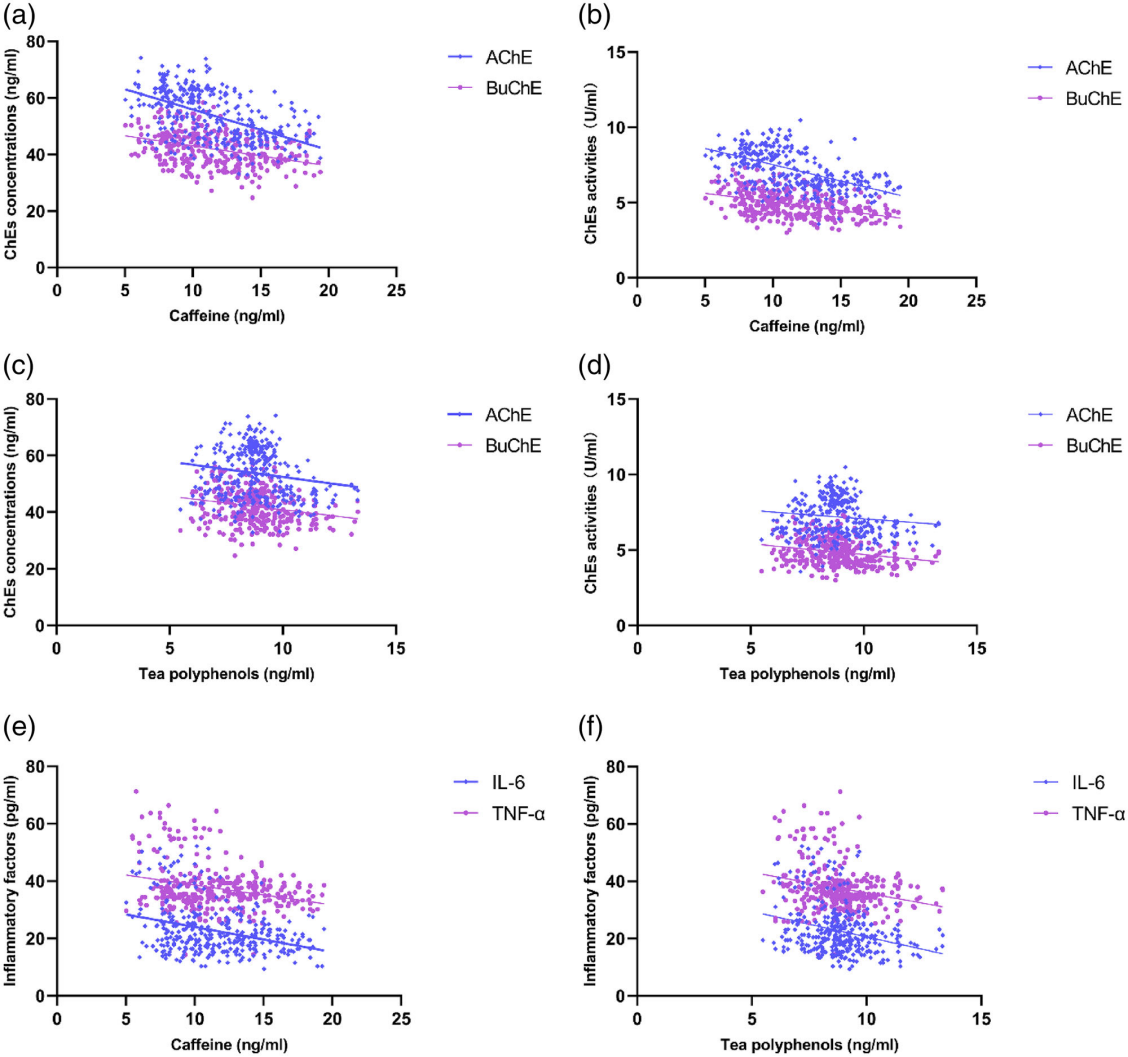
Correlations between preoperative plasma biomarkers of tea and other biomarkers. The scatter plots depict the associations of the concentrations of AChE (a), BuChE (a), IL‐6 (e), and TNF‐α (e) and the activities of AChE (b) and BuChE (b) at *T*_0_ with the concentrations of caffeine at *T*_0_, and the associations of the concentrations of AChE (c), BuChE (c), IL‐6 (f), and TNF‐α (f) and the activities of AChE (d) and BuChE (d) at *T*_0_ with the concentrations of tea polyphenols at *T*_0_. AChE, acetylcholinesterase; BuChE, butyrylcholinesterase; ChEs, cholinesterases; IL‐6, interleukin‐6; *T*_0_, before anesthesia; *T*_1_, 24 h after surgery; TNF‐α, tumor necrosis factor‐α.

Figure 3 showed that the concentrations of AChE (*r* = −.535, *P* < .01) and BuChE (*r* = −.369, *P* < .01) as well as the activities of AChE (*r* = −.566, *P* < .01) and BuChE (*r* = −.432, *P* < .01) at *T*_0_ were negatively associated with caffeine concentration at *T*_0_ (Figure 3a,b), while the concentrations of AChE (*r* = −.146, *P* = .008), BuChE (*r* = −.207, *P* < .01), IL‐6 (*r* = −.249, *P* < .01), and TNF‐α (*r* = −.200, *P* < .01) as well as the activities of AChE (*r* = −.110, *P* = .046) and BuChE (*r* = −.237, *P* < .01) at *T*_0_ were weakly associated with tea polyphenol concentration at *T*_0_ (Figure 3c,d,f). In addition, there were no significant correlations of caffeine concentration at *T*_0_ with the concentrations of IL‐6 (*r* = −.266, *P* < .01) and TNF‐α (*r* = −.168, *P* = .002) (Figure 3e).

## 4 DISCUSSION

In this prospective cohort study of Chinese elderly, we evaluated the association between habitual tea consumption and POD in 332 elderly adults who underwent hip/knee arthroplasty under combined spinal and epidural anesthesia. An increase of studies focused on the prediction and treatment of POD, while there was still a lack of evidence focused on correlations between POD and biomarkers of tea. Our study is to explore the impact of habitual tea consumption on POD from the perspective of dietary intake.

Aging and neurodegenerative diseases are well‐known risk factors for delirium (Inouye, 1996), and previous cognitive impairment is a major risk factor for POD (Davis et al., 2015). Preoperatively, we selected subjects aged 65–85 years, excluded subjects with cognitive impairment, and compared the MMSE scores of all subjects at baseline. Our data suggested that there was no significant difference in MMSE score at baseline between patients with and without POD. In this cohort study, we observed that the incidence of POD following total joint arthroplasty was 18.37% (*n* = 61 of the 332 patients, 5.72% in habitual tea consumers and 12.65% in infrequent tea consumers), which was largely consistent with two previous studies reporting a POD incidence of 16.5% and 17% (Chen et al., 2017; Scott et al., 2015). As expected, for those who had infrequent tea consumption, we found that they appeared to be more likely to develop POD (12.65%, *n* = 41 of the 332 patients), which indicated that habitual tea consumption might have an impact on the occurrence of POD. In regression analyses, we found that habitual tea consumption was significantly associated with POD after adjustment for age and ASA physical status. The adjusted OR value was 0.353 (*P* = .001), and the 95% CI was 0.190–0.655, which indicated that habitual tea consumption was negatively correlated with POD. That is to say, habitual tea consumption after adjustment is still associated with POD and it is a protective factor for reducing the occurrence of POD. Likewise, the same went for biomarkers of caffeine and tea polyphenols in plasma at *T*_0_ and *T*_1_ as well as in CSF.

CSF is in direct contact with the extracellular space of the brain, and it can reflect the biochemical changes that occur in the brain. Compared with biomarkers in plasma, CSF biomarkers have higher clinical significance and diagnostic value (Olsson et al., 2016). Therefore, CSF is the optimal source of POD biomarkers (Xie et al., 2014). Plasma is less invasive and more readily available, and spinal epidural block is a convenient method to collect CSF samples from subjects in our study. Because CSF could not be collected after operation, we compared and analyzed the preoperative biomarkers in plasma and CSF to verify their correlations so as to estimate their postoperative consistency. Spearman's rank correlation indicated that the concentrations of preoperative caffeine, tea polyphenols, AChE, and BuChE in plasma were significantly correlated with those in CSF. Additionally, the activities of preoperative AChE and BuChE in plasma were correlated with those in CSF. Consequently, there is a strong consistency of preoperative biomarkers in plasma and CSF, from which it can be inferred that biomarkers in CSF after operation may be also highly consistent with those in plasma.

For Chinese, especially the elderly, tea drinking is a particular dietary habit. Studies have found that drinking tea can improve mood, concentration, and mental performance (Gardner et al., 2007; Gilbert, 2019). Previous research indicated that tea consumption was significantly associated with cognitive impairment and mortality (Qiu et al., 2012; Ruan et al., 2013). All types of tea contain a large amount of caffeine and tea polyphenols. In our study, after excluding confounding variables such as age, gender, height, body weight, and BMI, we revealed that caffeine and tea polyphenols in plasma at *T*_0_ and *T*_1_ and in CSF were significantly associated with habitual tea consumption, which was consistent with our common knowledge about patients who had frequent tea consumption. Previous studies have confirmed that caffeine and tea polyphenols play important roles in prevention and intervention for cognitive decline (Ide et al., 2014; Park et al., 2011). Then, how does habitual tea consumption exert impact on neurodegenerative diseases such as POD?

To our knowledge, ACh is a classic neurotransmitter that is widely distributed in the nervous system and considered to play a key role in the regulation of learning and memory, especially in the regulation of cognitive function (Naicker et al., 2016). As mentioned above, cholinesterase inhibitors increase central ACh levels by prolonging the retention of ACh in the synaptic cleft, thereby enhancing cholinergic neurotransmission (Okello et al., 2012). It has been confirmed that the main treatment of cholinergic impairment is the use of cholinesterase inhibitors (Akomolafe et al., 2017). In our study, we mainly focused on the effects of habitual tea drinking on POD by analyzing the associations of cholinesterase with biomarkers of caffeine and tea polyphenols in the plasma at *T*_0_ among 332 patients. The concentrations of AChE and BuChE as well as the activities of AChE and BuChE at *T*_0_ were negatively associated with the concentrations of caffeine and tea polyphenols at *T*_0_, whereas these associations were relatively weak, which may be explained by the inhibition of cholinesterase caused by caffeine and tea polyphenols. Namely, higher concentrations of caffeine and tea polyphenols were associated with lower concentrations and activities of cholinesterase. Caffeine can inhibit cholinesterase in rat brain (Akomolafe et al., 2017), and tea polyphenols can inhibit AChE and BuChE, as well as enhance the cholinergic neurotransmission (Adsersen et al., 2006; Ali et al., 2016; Kulišić‐Bilušić et al., 2008), which is consistent with our results.

Peripheral inflammation can induce further cytokine release in cerebral tissue through pro‐inflammatory cytokines, leading to overactivated microglia and disrupted BBB. Furthermore, overactivated microglia and disrupted BBB create a neurotoxic response, cause neuronal injury, and affect neuronal function (Dantzer et al., 2008; Hughes et al., 2012; MacLullich et al., 2011). Neuroinflammatory factors, such as IL‐6 and TNF‐α, induce inflammation of the nervous system, which results in dysfunction of neurons and synapses and leads to neurobehavioral and cognitive symptoms of POD (Maldonado, 2013). Cholinergic anti‐inflammatory pathway that interrupts a vicious cycle of neuroinflammation plays an important part in inflammatory response on POD. In addition to improving disrupted BBB caused by TNF‐α and IL‐6, it is theoretically plausible that cholinergic anti‐inflammatory pathway prevents neuroinflammatory factors from affecting cognitive function due to entry via disrupted BBB (Evered et al., 2016). In this study, the concentrations of IL‐6 and TNF‐α at *T*_0_ were also negatively associated with the concentrations of caffeine and tea polyphenols at *T*_0_, which may be explained by the enhancement of the cholinergic anti‐inflammatory pathway induced by caffeine and tea polyphenols. However, there were weak correlations of the inflammatory factors (IL‐6 and TNF‐α) with caffeine and tea polyphenols. It is possible that inflammatory response has some special underlying changes in the vulnerable brain, which exerts an influence on correlation analysis in our study. A previous study found, when evaluating plasma and CSF samples for up to 30 h postoperatively, a consistent upregulation of CSF IL‐6 and undetectable TNF‐α was found. And IL‐6 level might correlate with sleep disorders (Buvanendran et al., 2006). Another study has also pointed out that the extent of inflammatory reaction is related to the degree of postoperative cognitive changes (MacLullich et al., 2011).

Probably, in POD patients, there are increased levels of neuroinflammatory factors and suppression of cholinergic nervous system. In other words, inhibiting neuroinflammation or activating the cholinergic nervous system may affect the development of POD, which may be due to changes in AChE, BuChE, and inflammatory factors as indicated by our research. Changes in contents and activities of cholinesterase, caused by habitual tea drinking, probably produce an effect on the cholinergic nervous system.

As for the differences in the associations of POD with the biomarkers of caffeine and tea polyphenols, we speculated that the differences were associated with the different intricate effects of caffeine and tea polyphenols on the central nervous system. ACh levels are regulated not only by the changed cholinergic nerve system but also by adenosine receptors that are combined with caffeine (Acquas et al., 2002; Van Dort et al., 2009).

Inevitably, several potential limitations of this study should be taken into account. First, the current pilot findings do not provide strong evidence that habitual tea consumption can affect the occurrence of POD due to the small sample size. Our results will need to be validated by a larger study. Second, the data on habitual tea consumption in our study were self‐reported. Although subjective evaluation of the frequency of tea consumption is likely to be important and valid, this type of measure is not standardized, which may limit comparability with other studies. The measurement for this self‐reported tea consumption may be a concern. However, we have little concern about the consistency of such a measurement in the findings because our results are based on the cohort study. Third, although all types of tea are rich in caffeine and tea polyphenols, the associations between various measures of tea consumption and POD, like the amount of tea leaves added, types of tea, and duration of tea consumption, have yet to be examined. Further research is needed to elucidate potential contributing factors to the pathogenesis of POD. Additional considerations include CSF and plasma sampling over stationary period to consider other than the effect of circadian variations.

In spite of these shortcomings, we have extended the existing research by providing a new estimate of the association between habitual tea consumption and POD among Chinese elderly. The results of this study suggest that habitual tea consumption is probably associated with lower incidence of POD compared with patients who had no tea drinking habit. Because tea is easily available and cheap and has no side effects at normal consumption level, drinking tea is potentially a good preventive measure for neurodegenerative diseases such as POD and may help to reduce healthcare cost during hospitalization. This is especially relevant in the context of a growing and fast‐aging world population.

## 5 CONCLUSION

Habitual tea consumption may be associated with a lower incidence of POD following knee/hip arthroplasty surgery in elderly patients, which may correlate with inhibition of cholinesterase by caffeine and tea polyphenols, as well as enhancement of cholinergic neurotransmission. To date, it remains unknown whether there are other potential contributing factors to the pathogenesis of POD, pending further investigations.

## CONFLICT OF INTEREST

The authors declare no conflict of interest.

### PEER REVIEW

The peer review history for this article is available at https://publons.com/publon/10.1002/brb3.2612

## Data Availability

All data generated or analyzed during this study are included in this published article.
